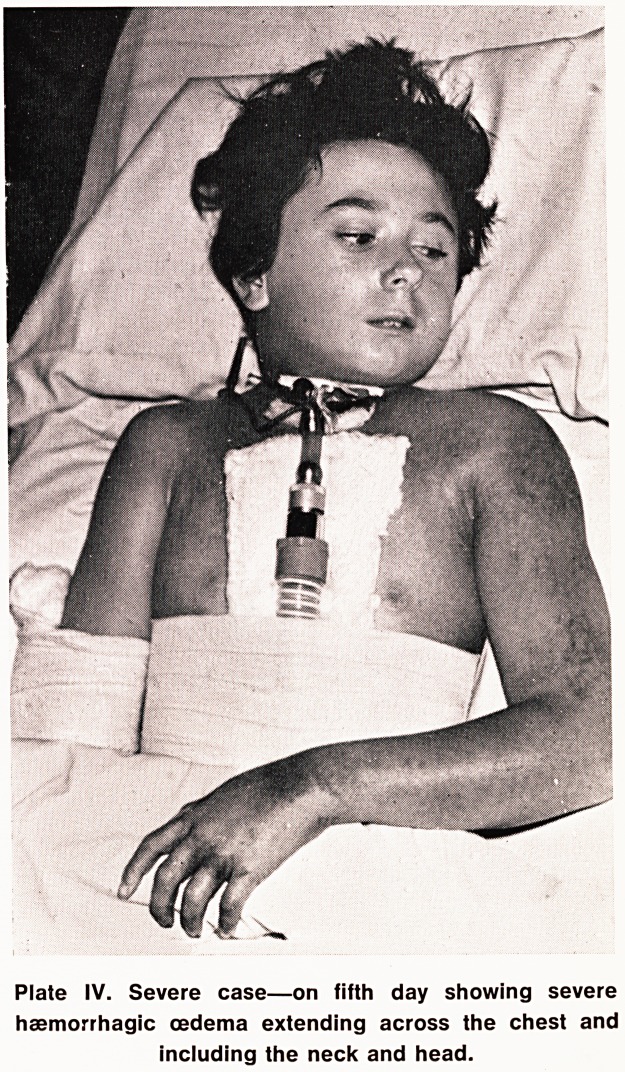# Vipers and Viper Bites in the West Country

**Published:** 1970-01

**Authors:** C. J. Moran

**Affiliations:** Medical Registrar, Ham Green Hospital, Bristol


### Vipers and Viper Bites in the West Country

C. J. Moran, B.Sc., M.B., Ch.B.
Medical Registrar, Ham Green Hospital, Bristol

Vipers are said to feel most at home in clearings, along the edges of woods, and on moors and mountains. The countryside around Bristol is often of this type and much favoured by our serpentine brothers, as anyone will agree who has walked in the Mendips and seen them dozing in the shade of notices reading: "Beware of Adders: "If bitten ring . . . " Priddy Pool is particularly recommended for an encounter of this kind.

Plate I. A viper on a Mendip picnic.

As is well known the "Viper berus" (Plate I) is our only poisonous snake and grows to about two feet in length. The much larger grass snake, "Natrix natrix", may reach five feet but is quite harmless. The worst it can do is to leave a very unpleasant smell on the hand of its aggressor. Nevertheless it is widely persecuted for the sins of its distant cousin. The only reliable colouring which distinguishes these two is the dark longitudinal zigzag down the viper's back which is fairly constant (Plate 1). Both may otherwise be a similar shade of grey.

The venom, from a modified salivary gland, is not very toxic as venoms go, that of the Russell viper being forty times more lethal to the average guinea-pig. It is not well described in the literature, but seems to consist principally of a little neurotoxin, and a larger quantity of haemorrhagin. This latter has an affinity for the endothelial cells of finer capillaries and produces a characteristic haemorrhagic oedema in man. The snake's natural prey (mice, frogs, rabbits, etc.) are probably paralysed by the one and "homogenized" by the other.

Mating takes place in April and May, the young being born in August and September.

### THE BITE

The season for bites extends from March to October, the reptile hibernating for the rest of the year. The clinical severity of a bite is thought to depend on the quantity of venom injected, and Morton (1967) believes this will depend on the time interval since the previous bite. In support of this he describes how there was a marked discrepancy in the severity of the local effects in a boy bitten on each hand in succession. Similarly a paratrooper was bitten and severely affected when he made overtures to a viper imprisoned in the warm cab of a lorry for two days without food. However, a five year old boy who provoked a hibernating adder and was bitten for his pains, escaped lightly, even though the beast had presumably not bitten for some weeks.

### RESULTANT DEATHS

Rightly or wrongly the general public seems to regard the viper as being little more dangerous than the bee. Walker (1945), reviewing deaths in England, described seven up to 1945. Antivenom was not available at this time and so the clinical picture was uncomplicated, death being due to circulatory collapse and unconsciousness within six to thirty-six hours. In the last twenty years the only recorded cases in the U.K. are a boy who died of allergy to the antivenom in 1957 (Lancet 1957) and our own case in 1961.

### REVIEW OF CASES AT HAM GREEN HOSPITAL 1960-69

Until recently Ham Green Hospital was a centre designated to hold the Pasteur Institute E.R. serum. However, this has not been in regular usage for some seven or eight years since its efficacy was never proved, and, being a horse serum against the venom of a South African viper, "Cerates cornutus", often seemed to do more harm than good. Nevertheless, the fact that antivenom was available attracted cases from a considerable area, totalling 10 in as many years. Presumably there are very many more cases ignored by the victim or treated by local practitioners which never come to hospital.

### MILD CASES

Four of our cases had no general symptoms and only minimal local reaction. They were males aged nine, ten, fourteen and forty-six years, bitten on the ankles and feet. There was only a little swelling round the puncture marks.

Two more mild cases came from Shipham. A five year old girl was bitten on the foot, and gross swelling of the whole leg gradually developed. There was marked bruising along its length, still very obvious on her discharge eight days later.

The other, a twenty-four year old teacher was bitten on the hand. Hæmorrhagic swelling of the arm became very severe, making it very tense and tender despite treatment with antihistamines and steroids.

A nineteen year old nurse from Porlock had mild swelling and, before she arrived was treated with anti-venom without adverse effect, though claiming to be allergic to bee stings and formalin vapour.

### MORE SEVERE CASES

The first case with systemic effects was an eleven year old boy from Axbridge in June 1960. After a bite on the thumb he began to sweat and, within fifteen minutes, developed abdominal pain and vomiting. Epic measures on the spot, half an hour later, included the use of a tourniquet and lancing the thumb, but in spite of this he was able to walk into hospital. On examination his blood pressure was down to 70 mm Hg. systolic, and he was cyanosed. There was gross swelling of the arm which increased for two days. He continued to vomit and antivenom was given. Ten days later he was discharged home to continue his main hobby of collecting vipers. This specimen would have been his twentieth !

### A FATAL CASE

The most unhappy case occurred in May 1961. A healthy twelve year old girl was picnicking with the family by Priddy Pool in the Mendips. During a final stroll before leaving she suddenly ran to the nearest bystander saying she had just been bitten by a snake. She began to vomit and within a few minutes collapsed, breathing heavily, with cyanosis and swelling of the face and lips. Within half an hour she was given an antihistamine injection and then rushed by ambulance with police escort to Ham Green Hospital, where she arrived two hours after the bite occurred. At this time she was extremely restless and disorientated. The blood pressure became unrecordable, and the pupils fixed and dilated. Paraldehyde was given as sedation but physical restraint was needed for the rest of her stay. Fang marks were clearly visible over the lateral malleolus, bruising was beginning to spread up the leg (Plate II), and there was bloody diarrhœa and intermittent vomiting.

Energetic resuscitation with intravenous fluids, hydrocortisone, adrenaline and noradrenaline produced a temporary improvement. The blood pressure rose and pupil responses became normal. However, she deteriorated and died eighteen hours later. She had been given 2.5 litres of fluid, but antivenom was avoided. The hæmoglobin level was 82%. Post mortem examination showed a very widespread hæmorrhagic reaction in almost all parts of the body. On careful dissection the fang marks were shown to be well away from any significant vein.

**Plate II. The fatal case—showing fang marks (ringed) and bruising around.**

### A SEVERE CASE

A happier but equally dramatic case presented in August of 1969. A healthy ten year old girl and her family were motoring along the A38 to Birmingham after a West Country holiday. At Churchill they stopped the car to pick blackberries. On stooping to pick up some fallen fruit from the hedge bottom, she saw the snake as it struck, stood up and then became dizzy and slid down the bank on to the road. Her brother came to her side and clearly saw the snake making good its escape. She claims not to have been frightened but her condition quickly began to deteriorate despite an injection of piriton wisely given by a local practitioner.

On arrival at Ham Green Hospital one and a half hours later, she was unresponsive with cold extremities and fixed, dilated pupils. Pulse was 120/minute and blood pressure 70 mm Hg. systolic. Intravenous therapy was quickly instituted, but before it could begin she suddenly recovered, her blood pressure increased to 90/70 mm Hg. and pupil reaction became normal. She was able to answer questions in between bouts of diarrhœa and vomiting. This dramatic improvement in her condition unfortunately proved to be only the lull before a week long storm. Thirty-six hours after admis-

**Plate III. Severe case—early swelling and bruising of left arm.**

**Plate IV. Severe case—on fifth day showing severe hæmorrhagic œdema extending across the chest and including the neck and head.**

sion she again deteriorated with disorientation and hypotension.

The saga of her subsequent progress is best understood in retrospect by highlighting two main features. The first was great restlessness and agitation necessitating physical restraint and constant sedation. The second factor was the gross haemorrhagic oedema. This spread up the arm eventually to include the chest, neck and head, completely masking her normal shape and body contours (Plates III and IV). It clearly contained very large quantities of blood and fluids. Her hæmoglobin of 105% initially, fell to 32% over five days. The blood pressure frequently fell to below 80 mm systolic despite monitoring central venous pressure and giving many litres of saline. Serum proteins fell to 4.3 G/100 ml. On the fourth day, œdema of the neck and presumably inside the thorax combined with sedation to cause respiratory difficulties and sternal recession. A tracheotomy was performed.

The situation was not set to rights until plasma and blood transfusion were given, when the blood pressure rose and her clinical condition quickly improved on the fifth and sixth days. Estimations of serum and urine electrolytes were consistent with the passage of large quantities of plasma protein, fluid and electrolytes into the tissue spaces. Bleeding, clotting and prothrombin investigations were normal and there was no evidence of hæmolysis in the serum. Electrocardiograms were also normal.

This clinical story is similar in many ways to that of the paratrooper mentioned above who also revived from a moribund state only to deteriorate again thirty hours later. This patient was described by Brown and Dewar (1965), since he was unique in developing electrocardiographic changes and heart failure consistent with a myocardial infarction.

### IN CONCLUSION

If one is ever to enjoy the Mendips again it clearly needs to be emphasized that the above cases are exceptional. The vast majority of bites produce only mild poisoning and local reaction. When moderate poisoning occurs, local reaction may be more intense, and the whole limb be involved with swelling and hæmorrhagic discoloration. General symptoms occur within the first hour, sweating, diarrhœa and vomiting with abdominal pain, presumably caused by the neurotoxin. There may be an acute episode of unconsciousness or semiconsciousness which quickly reverts, possibly with cardiovascular collapse. In severe cases the shocked state persists and needs intensive supportive treatment with blood transfusion.

The balance is well set by a recent leading article (Lancet 1969) which emphasizes that only a minority of human victims receive enough venom to cause serious poisoning. It goes on to deplore enthusiastic first-aid measures such as tourniquets and lancing. Usually reassurance, rest and possibly antihistamine injection is all that is required before the patient is moved to hospital.

In hospital sedation is best achieved with paraldehyde. Antibiotics, steroids and tetanus prophylaxis are not indicated.

Antitoxin formed from the European long nosed viper is said to be very effective against V. berus, but it seems only to be available from Yugoslavia at present! Certainly there is as yet none available in the West Country. From our own experience we would recommend that blood transfusion be considered early in the severe cases.

**Acknowledgements**

I wish to thank Dr. J. Macrae and Dr. R. V. Walley for permission to describe their cases.

### REFERENCES

**Brown R. and Dewar H. A.** (1965) *Brit. Heart J.* **27,** 144.
Lancet 1957, **1,** 1095. (Annotation).
Leading Article, *Lancet* 1969, **3,** 370.
**Morton T. C. (1967)** *Brit. med. J.* **2,** 373.
**Walker C. W. (1945)** *Brit. med. J.* **2,** 13.

## Figures and Tables

**Plate I. f1:**
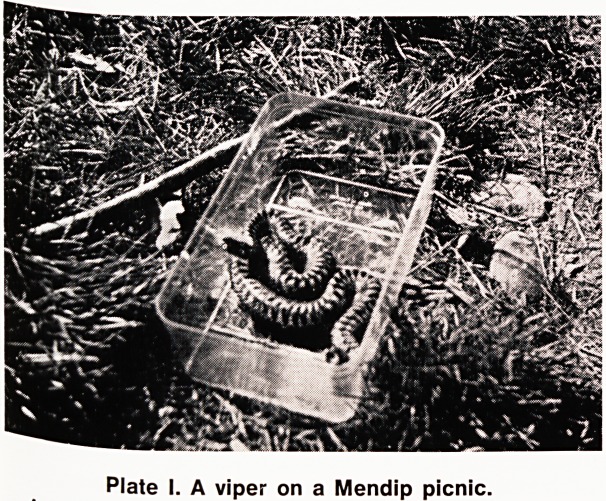


**Plate II. f2:**
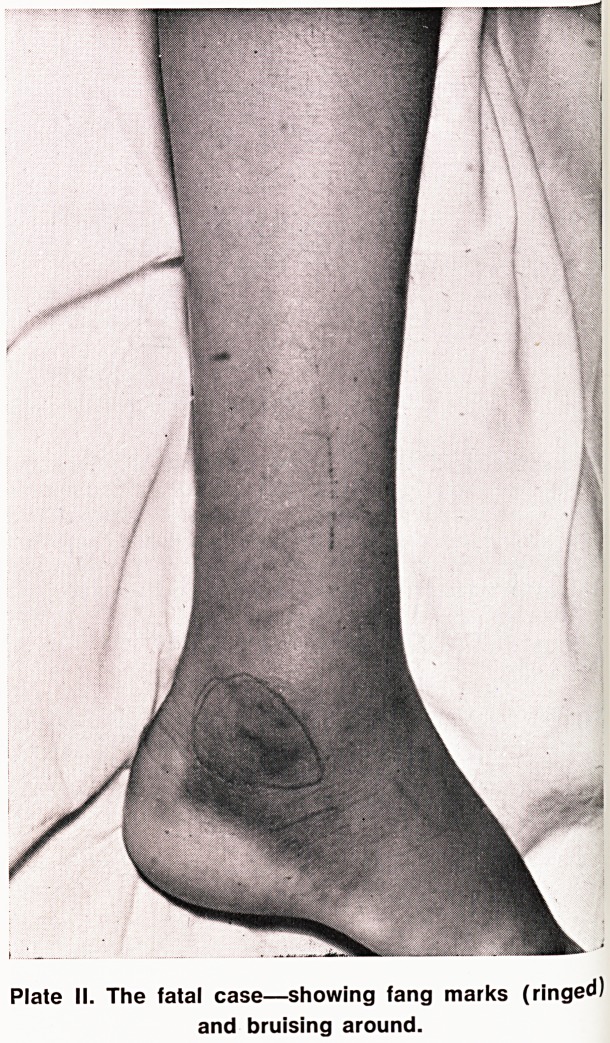


**Plate III. f3:**
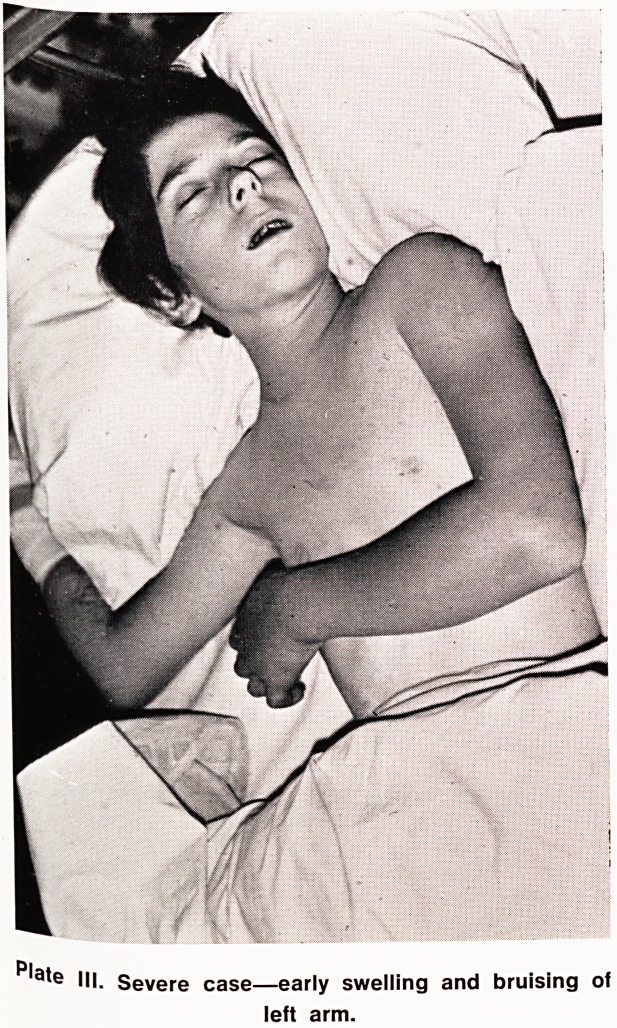


**Plate IV. f4:**