# Vipers and Viper Bites in the West Country

**Published:** 1970-04

**Authors:** 


### CORRECTION

In the article, "Vipers and Viper Bites in the West Country" by C. J. Moran published in this Journal Vol. 85, p. 6 (1970), reference is made to Leading Article, Lancet 1969, **3,** 370. This should have read Leading Article, Brit. med. J. 1969, **3,** 370. We apologise for the error.

